# Better reporting for better research: a checklist for reproducibility

**DOI:** 10.1186/s13742-015-0071-8

**Published:** 2015-07-23

**Authors:** Amye Kenall, Scott Edmunds, Laurie Goodman, Liz Bal, Louisa Flintoft, Daniel R Shanahan, Tim Shipley

**Affiliations:** 1 BioMed Central, London, United Kingdom; 2 BGI, Hong Kong, Hong Kong; 3 Genome Biology, BioMed Central, London, United Kingdom; 4 BMC Neuroscience, BioMed Central, London, United Kingdom

*How easy is it to reproduce or replicate the findings of a published paper?* In 2013 one researcher, Phil Bourne, asked just this. How easy would it be to reproduce the results of a computational biology paper? [1]. The answer: 280 hours. Such a number is surprising, given the theoretical reproducibility of computational research and given Bourne was attempting to reproduce work done in his own lab. Now at the National Institutes of Health (NIH) as Associate Director of Data Sciences, Bourne is concerned with the reproducibility of all NIH funded work, not just his own—and the problem is large. In addition to work in computational biology (which theoretically should be more easily reproducible than “wet lab” work), hallmark papers in cancer through to psychology have been flagged as largely unreproducible [2, 3]. Closer to home, *GigaScience* has carried out similar work to quantify reproducibility in their content. Despite being scrutinized and tested by seven referees, it still took about half a man-month worth of resources to reproduce the results reported in just one of the tables [4]. “Reproducibility” is now increasingly on the radar of funders and is making its rounds in the wider media as well, with concerns of reproducibility making headlines at *The Economist* [5] and *New York Times* [6], amongst other outlets.

## Why is this important?

It is critical to note that irreproducible work doesn’t necessarily mean fraud occurred, nor even that the findings are incorrect; likewise, reproducible research can still be incorrect. While this key point is well-understood by most scientists, this is not always easy to explain to the general public. However, as most research is paid for through tax payers, public trust in research is essential. We—researchers, funders, and publishers—must do a better job at communicating this message to the public. We must better explain that science is an activity that continually builds on and verifies itself. But we also must develop policies that better support this process—policies, for example, that promote transparency and allow for improved verification of research.

Clearly important for clinical research, verification is equally important for preclinical research, something we all have an equal stake in. No one can innovate new drugs overnight, no matter how rich they are, no matter which doctor they see. Better, more robust preclinical research benefits us all.^1^ Our ability to rely on published data for potential therapeutics is critical, and recently its reliability has been called into question [7].

One well-publicised example of this was brought to light in an oncology study of preclinical research findings in which researchers were able to confirm only 11 % of the findings [8, 9]. Although the relevance of more robust research is clear in the area of oncology, it is also important for more exploratory research that might never make it to the preclinical setting. Funding and time are both increasingly limited, and the waste generated from follow-up work based on irreproducible research is high. A recent study by Freedman et al. estimated this at approximately $28 billion a year for preclinical research in the United States alone [10].

## Funder update

The NIH have recently taken bold steps to begin to tackle the need for better design, more appropriate analysis, and greater transparency in the conduct and reporting of research. In January 2014 the NIH announced they would fund more training for scientists in data management and restructure their grant review process to better value other research objects, such as data [11]. But it is peer review and the editorial policies and practices of journals that have come under the greatest scrutiny, and in June 2014 a set of guidelines for reporting preclinical research were proposed by the NIH to meet the perceived need for more stringent standards [12]. These guidelines ask journals to ensure, for example, that authors have included a minimum set of information on study design, that statistical checks have been carried out by reviewers, and that authors have been given enough information to enable animal strains, cell lines, reagents, and so on, to be uniquely identified reagents. (For a full list of requirements, see the NIH Principles and Guidelines for Reporting Preclinical Research.)

## BioMed central author and reviewer checklist

Journals clearly have an important part to play in helping to ensure as far as possible that experimental design and analysis are appropriate, and that reporting standards are met. This month BioMed Central will launch a trial checklist for authors and referees with these explicit aims.

BioMed Central has long supported transparency in reporting for both biology and medicine, even working with Editorial Board Members developing and endorsing standards such as MIQE-precis [13], and the EQUATOR Network guidelines, such PRISMA [14]. The trial checklist builds on these accepted standards and the principles behind them, formalising, tailoring and standardising these efforts across journals.

The checklist addresses three areas of reporting: experimental design and statistics, resources, and availability of data and materials [15]. Some of the NIH Guidelines were straightforward to implement, given they were policies long in place at BioMed Central. However, we used these new guidelines as an opportunity to ensure that these as well as our long-standing policies already in place had the best chance of being adhered to by authors and by reviewers by integrating them into our internal systems and workflows. Authors will be asked on submission to confirm that they have included the information asked for in the checklist or give reasons for any instances where it is not made available or not applicable.^2^ Likewise, reviewers will be asked to confirm the information has been satisfactorily reported and reviewed.

This also has the aim of making editors’ jobs more straightforward. With a clear and simple checklist on what information to include in the manuscript, less time should be spent liaising with authors. Plans are also in place to integrate our new checklist into BioMed Central Roadshows and Author Workshops (http://roadshow.biomedcentral.com/), helping to ensure researchers are made aware of the reporting standards *before* publication.

BioMed Central is not the first to implement reporting guidelines, with the Center for Open Science [16]^3^ and our colleagues at *Nature*. [17] also recently announcing similar initiatives. Implementing reporting guidelines, rather through a checklist or another means, is not simple. Exploratory research that does not have the immediate practical implications of preclinical research often does not easily adhere to the criteria of reproducibility. For this reason we are implementing this first as a trial, for which we will collect feedback and monitor its success.

In the first instance, the checklist will be rolled out on a small group of select journals: *BMC Biology*, *BMC Neuroscience*, *Genome Biology*, and *GigaScience*. In 6 months’ time, we plan to review the data we have collected around this trial, checking whether reporting has increased and collating author, editor, and reviewer feedback on the trial, with the aim to roll out the checklist (with any revisions) across all BioMed Central journals. We have designed the checklist to act as an aid to authors, editors, and reviewers rather than a burden to submission and look forward to hearing your thoughts as the trial progresses.

## Footnotes

1 For further discussion of this around clinical trial transparency and reliability, see Ben Goldacre’s *Bad Pharma.*

2 To better support our authors in adhering to this checklist, we have also recently revised our section on data availability, detailing where authors can deposit their data and how to cite their data in their manuscript. We also have in-house staff available to work with authors to find a home for their data. http://www.biomedcentral.com/about/editorialpolicies#DataandMaterialRelease

3 The Center for Open Science with stakeholders from research have recently devised an easy to use set of guidelines based on eight standards and three levels of adherence. With this checklist, all journals will adhere to level 2 requirements. At present, all BioMed Central journals adhere to level 1 requirements. http://www.sciencemag.org/content/348/6242/1422.figures-only
